# Re-engineering a neuroprotective, clinical drug as a procognitive agent with high in vivo potency and with GABA_A_ potentiating activity for use in dementia

**DOI:** 10.1186/s12868-015-0208-9

**Published:** 2015-10-19

**Authors:** Jia Luo, Sue H. Lee, Lawren VandeVrede, Zhihui Qin, Sujeewa Piyankarage, Ehsan Tavassoli, Rezene T. Asghodom, Manel Ben Aissa, Mauro Fà, Ottavio Arancio, Lan Yue, David R. Pepperberg, Gregory R. J. Thatcher

**Affiliations:** Department of Medicinal Chemistry and Pharmacognosy, University of Illinois College of Pharmacy, UIC, 833 S. Wood St., Chicago, IL 60612-7231 USA; Department of Pathology and The Taub Institute for Research on Alzheimer’s Disease and the Aging Brain, Columbia University, 630 W 168th St., New York, NY 10032 USA; Department of Ophthalmology and Visual Sciences, Illinois Eye and Ear Infirmary, Lions of Illinois Eye Research Institute, University of Illinois at Chicago, 1855 W. Taylor St., Chicago, IL 60612 USA; Department of Bioengineering, University of Illinois at Chicago, 851 S. Morgan St., Chicago, IL 60607 USA; Department of Ophthalmology, University of Southern California, 1441 Eastlake Ave., Los Angeles, CA 90033 USA

**Keywords:** Alzheimer’s disease, CMZ, Chlormethiazole, NMZ, sGC/NO/cGMP, pCREB, LTP, Cognitive deficits

## Abstract

**Background:**

Synaptic dysfunction is a key event in pathogenesis of neurodegenerative diseases such as Alzheimer’s disease (AD) where synapse loss pathologically correlates with cognitive decline and dementia. Although evidence suggests that aberrant protein production and aggregation are the causative factors in familial subsets of such diseases, drugs singularly targeting these hallmark proteins, such as amyloid-β, have failed in late stage clinical trials. Therefore, to provide a successful disease-modifying compound and address synaptic dysfunction and memory loss in AD and mixed pathology dementia, we repurposed a clinically proven drug, CMZ, with neuroprotective and anti-inflammatory properties via addition of nitric oxide (NO) and cGMP signaling property.

**Results:**

The novel compound, NMZ, was shown to retain the GABA_A_ potentiating actions of CMZ in vitro and sedative activity in vivo. Importantly, NMZ restored LTP in hippocampal slices from AD transgenic mice, whereas CMZ was without effect. NMZ reversed amnestic blockade of acetylcholine receptors by scopolamine as well as NMDA receptor blockade by a benzodiazepine and a NO synthase inhibitor in the step-through passive avoidance (STPA) test of learning and working memory. A PK/PD relationship was developed based on STPA analysis coupled with pharmacokinetic measures of drug levels in the brain: at 1 nM concentration in brain and plasma, NMZ was able to restore memory consolidation in mice.

**Conclusion:**

Our findings show that NMZ embodies a promising pharmacological approach targeting synaptic dysfunction and opens new avenues for neuroprotective intervention strategies in mixed pathology AD, neurodegeneration, and dementia.

**Electronic supplementary material:**

The online version of this article (doi:10.1186/s12868-015-0208-9) contains supplementary material, which is available to authorized users.

## Background

Of age-related dementia, the most common form, Alzheimer disease (AD) a devastating neurodegenerative disorder, presents a severe economic and social burden worldwide [1]. In the rare, familial form of AD (~1 % of cases) [2], genetic mutations in specific proteins, *APP*, *PS1*, and *PS2*, cause insoluble amyloid-β peptide (Aβ) aggregation and elevated levels of neurotoxic, soluble Aβ_1–42_ [3–5]. In sporadic, late-onset AD, the appearance of Aβ neuropathology has supported the theory of Aβ deposition as the primary causative factor leading to neuronal death [6]. This hypothesis has resulted in the dominance in AD in drug discovery of transgenic mouse models that overexpress the mutant human genes linked to familial AD; however, to date, all therapeutics directly targeting Aβ production, aggregation, or clearance have failed primary endpoints in Phase 3 clinical trials [7–11].

Approximatively, half of the patients diagnosed with dementia, have additional pathologies associated to the hallmarks of Aβ and tau, including vascular dementia, and dementia with Lewy bodies [12, 13]. It is also increasingly recognized that AD and dementia are multifactorial diseases, with contributions to onset and progression including early synaptic failure [14], inflammation [15], oxidative stress [16], mitochondrial dysfunction [17], cerebrovascular impairment [18, 19], depletion of neurotrophins [20, 21], and excitotoxicity [22]. It is argued that effective treatment should target two or more factors in disease pathogenesis [23]. In this context, drug repositioning is also an attractive approach [24]. The clinical agent, chlomethiazole (CMZ), is neuroprotective in animal models [25], with reported amelioration of inflammation, mitochondrial dysfunction, and excitotoxicity [26–28]. Therefore, CMZ was proposed as a candidate for combination therapies treating neuronal injury [29, 30]. In line with this postulate, we have reported that CMZ is neuroprotective in primary neurons treated with neurotoxic, oligomeric Aβ_1–42_ (oAβ) [31].

Based on the strong a rationale for targeting synaptic dysfunction in AD [32], we sought to modify CMZ to incorporate this activity without loss of other attributes. Phosphorylation of cAMP-response element binding protein (CREB) is necessary for memory formation and synaptic strengthening and the work of Kandel has highlighted a role for nitric oxide induced release of cyclic guanosine 3,5-monophosphate (cGMP) in CREB activation in the hippocampus [33–35]. More recently decreased activity of pCREB has been demonstrated in homogenates taken from post-mortem AD patients [36]. Moreover, dysfunctional CREB signaling has been placed at the center of AD-related gene networks [37]. Accordingly, an analogue of CMZ, 4-methyl-5-(2-(nitrooxy) ethyl) thiazol-3-ium chloride (NMZ), was engineered to activate NO/cGMP/CREB signaling and to retain the beneficial activity of CMZ.

CMZ possesses anticonvulsant, anxiolytic, and sedative properties that derive, at least in part, from potentiation of GABA signaling, which has been confirmed by direct study of the actions of CMZ on GABA receptors [38, 39]. We successfully confirmed the retention of NMZ of these proprieties precisely, via potentiation of the α_1_β_2_γ_2_ GABA_A_ receptor and through study of sedative actions in vivo. Restoration of LTP was designed into the structure of NMZ and therefore comparison of NMZ and CMZ in hippocampal slices from the transgenic APP/PS1 mouse was used to confirm that the design of NMZ did add function over CMZ. Several agents have been reliably used to induce amnesia in wild type mice, which can be tested using the step-through passive avoidance (STPA) task. NO/cGMP signaling regulates the strength of synaptic transmission in an activity-dependent manner in the hippocampus and functions in both pre- and postsynaptic neurons [35, 40], therefore NMZ was tested using agents that induce amnesia by blockade of cholinergic, glutamatergic, and nitrergic signaling. Correlation of pharmacokinetic measurements of brain and plasma concentrations of NMZ with pharmacodynamic analysis of cognitive function in the face of scopolamine-induced amnesia were used to define PK/PD relationships.

Re-engineering of a neuroprotective agent that addresses multiple mechanisms contributing to neurodegeneration to provide a brain bioavailable small molecule that restores LTP and memory is a novel approach towards treatment of age-related and mixed pathology dementia, including the most common form, AD.

## Results

### NMZ is a GABA_A_ potentiator and shows a sedative effect at higher doses than CMZ

**Fig. 1:**
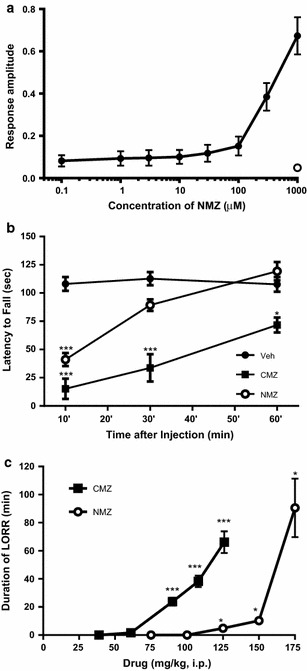
Retention of GABA_A_ potentiating, and attenuated sedative activity in NMZ relative to CMZ. **a** Oocytes expressing the α_1_β_2_γ_2_ GABA_A_ receptor (n = 6) showed a dose response to increasing concentrations of NMZ in the presence of GABA (6 µM). Addition of picrotoxin (200 µM) caused 96 ± 2 % inhibition of the potentiated GABA response (n = 4) (O). Data show mean ± SD normalized to the saturated 200 µM GABA response. **b** Male C57Bl/6 mice (n = 5–16) were injected i.p. with CMZ (45 mg/kg) or an equimolar dose of NMZ before testing for their latency to fall on a rotating rod (RR). NMZ showed less sedation than CMZ at various time points. Data show mean ± SEM. Statistical significance relative to vehicle is indicated by *p < 0.05, **p < 0.01, ***p < 0.001, using one-way ANOVA with Dunnett’s post hoc test. **c** Male C57BL/6 mice (n = 4–5) were injected with escalating doses of CMZ and NMZ and loss of righting reflex (LORR) was measured over 2 h. NMZ showed less sedation than CMZ, and no LORR was observed for NMZ until 125 mg/kg. Data show mean ± SEM. Non-zero statistical significance by one-sample t test is indicated by *p < 0.05, ***p < 0.001

The core anticonvulsant, sedative, and anxiolytic properties of CMZ derive, at least in part, from GABA potentiation. Measurements using the *Xenopus* oocyte model expressing α_1_β_2_γ_2_ GABA_A_ receptors confirmed that NMZ retained GABA_A_ potentiating activity in vitro (Fig. 1a). As a measure of sedation, motor impairment was assessed using latency to fall on a rotating rod for C57BL/6 mice (Fig. 1b). Preliminary experiments showed a significant effect of CMZ (50 mg/kg i.p.), therefore, this and an equimolar dose of NMZ were selected. All animals were trained in the task for 3–5 days prior to experiment, until latency to fall reached 100 ± 10 s. At 10 min post injection, CMZ and NMZ groups had a latency to fall of 14.2 ± 9.8 and 38.7 ± 5.4 s respectively (p < 0.001), showing motor impairment over vehicle treated animals (108.1 ± 6.2 s). At 30 min, only CMZ showed a significant deficit (35.0 ± 12.8 s; p < 0.001), versus vehicle (115.8 ± 6.3 s; NMZ 93.5 ± 5.4 s), and at 60 min, CMZ retained significant sedative actions (73.0 ± 6.5 s; p < 0.05; vehicle 122.1 ± 10.7 s; NMZ 109.1 ± 7.0 s). To test more profound sedation, loss of righting reflex (LORR) was assessed in male C57BL/6 mice (Fig. 1c). The duration of LORR, defined as failure to place four paws on the ground within 30 s after placing the mouse on its back, was measured every 2 min for 2 h post drug administration. All animals recovered fully after treatment. CMZ significantly induced LORR at doses lower than NMZ. At the 125 mg/kg dose, NMZ induced LORR for 4.8 ± 1.7 min, which was not statistically significant; whereas CMZ at a molar equivalent dose induced a significant LORR (23.8 ± 2.7 min). Only at the highest dose (175 mg/kg) did LORR reach significance for NMZ (LORR = 90.5 ± 20.9 min, compared to a molar equivalent dose of CMZ: LORR = 66.2 ± 7.7). It is important to note that the dose of NMZ needed to induce a transient sedative effect, reflected by LORR, was almost 200-fold higher than the procognitive dose reported below.

### NMZ reverses memory deficits in WT animals by consolidating memory

**Fig. 2:**
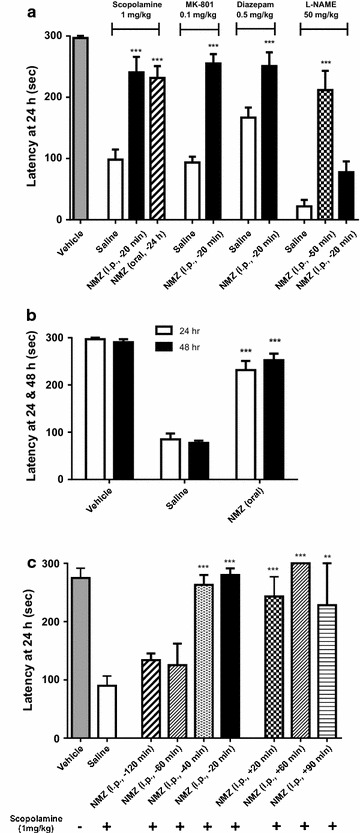
Reversal of induced amnestic deficits by multiple agents in STPA. **a** Male C57BL/6 mice (n = 5–10) treated with vehicle or NMZ (1 mg/kg, single dose i.p.; or 20 mg/kg/day, oral drinking water) after being administered with diverse amnestic agents were tested for their latency to enter the *dark side* of the STPA apparatus at 24 h after training. NMZ reversed memory deficits induced by scopolamine (1 mg/kg), MK-801 (0.1 mg/kg), diazepam (0.5 mg/kg), and L-NAME (50 mg/kg). **b** Male C57BL/6 mice with scopolamine-induced deficits treated with NMZ showed reversal of latency to enter the dark side of the chamber 24 and 48 h after training. Statistical significance is indicated by ***p < 0.001 compared with the respective saline control for each time point, using unpaired t test. **c** In scopolamine induced deficits, NMZ demonstrated reversal of cognitive deficits when administered i.p. between 40 min prior to training (−40 min) and 90 min after training (+90 min); but no significant effect was observed when administered 60 or 120 prior to training. Data show mean ± SEM. Statistical significance is indicated by *p < 0.05, **p < 0.01, ***p < 0.001 compared with the respective saline control for each treatment, using one-way ANOVA with Dunnett’s post hoc test

NMZ was designed to add procognitive activity to the neuroprotective actions of CMZ, therefore, NMZ (1 mg/kg) was tested in male C57BL/6 mice using the step-through passive avoidance (STPA) behavioral task, in which mice learn to associate a mild electric shock (0.5 mA) with the dark side of a light–dark box and latency to enter is assessed 24 and 48 h after training. A variety of amnestic agents, administered i.p. 30 min before training, have been shown to cause loss of memory without hindering the ability of animals to learn the task. The muscarinic receptor antagonist scopolamine (1 mg/kg) [41], the NMDA receptor antagonist MK-801 (0.1 mg/kg), the benzodiazepine diazepam (0.5 mg/kg) [42], and the nitric oxide synthase (NOS) inhibitor L-NAME (50 mg/kg), were able significantly to inhibit memory as reflected by decreased latency in STPA (Fig. 2a). Vehicle treated animals reliably show a latency in testing at or close to the cutoff threshold of 300 s, which was reduced after treatment with amnestic agents. Administration of NMZ (1 mg/kg i.p.) 20 min prior to training restored memory as shown by significantly increased latency (scopolamine 98.1 ± 16.6 vs. 240.7 ± 25.3 s treated; MK-801 94.6 ± 9.3 vs. 255.1 ± 15.4 s treated; diazepam 166.7 ± 16.6 vs. 250.9 ± 22.5 s treated; L-NAME 22.0 ± 10.4 vs. 211.7 ± 31.4 s treated 50 min prior to training). To test the effects of oral drug delivery, animals were treated with NMZ (20 mg/kg) in drinking water for 24 h prior to training, again resulting in reversal of a scopolamine-induced deficit, tested at 24 h (231.5 ± 19.3 s), or 48 h post training (Fig. 2b).

In the STPA task, animals are tested at least 24 h after drug administration, therefore, confounding locomotor drug effects are highly improbable. The task is also suited to correlate pharmacokinetics with pharmacodynamics (PK/PD). Using scopolamine-induced amnesia, the time of administration of NMZ (1 mg/kg i.p.) was varied from 120 min prior to 90 min post training (Fig. 2c). NMZ was procognitive when administered within 40 min prior to the start of training, while not effective when given earlier (−120 min: 134.0 ± 11.3 s; −60 min: 125.2 ± 37.1 s; −40 min: 263.0 ± 17.1 s; −20 min: 280.0 ± 11.4 s). When administered after training, the procognitive effect was significant at least up to 90 min (+30 min: 243.0 ± 34.0 s; +60 min: 300.0 ± 0.0 s; +90 min: 228.1 ± 71.9 s). Task acquisition during training did not differ significantly between vehicle and NMZ treated animals (data not shown).

### NMZ is orally bioavailable in the brain

**Fig. 3:**
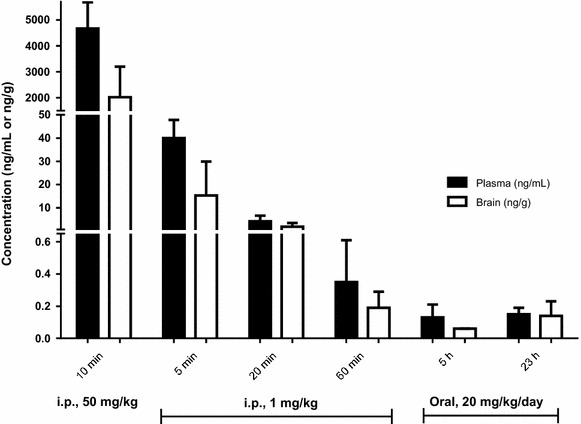
Pharmacokinetics study on male C57BL/6 mice treated with NMZ, single dose (50 or 1 mg/kg, i.p.) or supplied in hydrogel (20 mg/kg/day), showing bioavailability in brain and plasma at various time points after initiation of treatment. The estimated t_½_ for i.p. administration is 10 min

The loss of procognitive activity when NMZ is administered >60 min before training is indicative of drug clearance and a T_1/2_ < 60 min in mice. The plasma and brain concentrations of NMZ and its denitrated metabolite, 5-(2-hydroxyethyl)-4-methylthiazole (HMZ), were measured by LC–MS/MS (Fig. 3a), since HMZ is itself known to have bioactivity in vivo [43]. At early time points after bolus injections of NMZ at sedating (50 mg/kg i.p.) or procognitive doses (1 mg/kg i.p.), NMZ brain concentrations of 10.7 μM and 81.3 nM were measured, respectively. In STPA, NMZ is procognitive when administered 20 min prior to training, but loses activity administered 60 min prior to training: corresponding to brain levels of NMZ of 10.2 and 1.01 nM respectively. However, we also observed that NMZ administration prior to training is not required to restore memory, but that NMZ functions by consolidating memory when administered after training. The brain concentrations of NMZ and HMZ 5 h after injection were only 0.32 and 5.7 nM, respectively. In place of drug delivery in drinking water, drug was delivered in hydrogel that mice readily consume for hydration when drinking water is made unavailable. This delivery method achieves a relatively constant drug concentration throughout the awake-period, simulating an extended release clinical formulation [44]. Oral administration of NMZ (20 mg/kg) over 24 h, representing a procognitive dose in STPA, resulted in brain concentrations of NMZ and HMZ of 0.73 and 3.41 nM, respectively. Under these conditions, brain and plasma concentrations were not significantly different. Taken together, these measurements indicate that the brain concentration of NMZ required for memory consolidation after amnestic insult is approximately 0.5–1.0 nM. The measured concentration of HMZ could be used as a surrogate for the maximum theoretical concentration of NO released from NMZ, which would be 3–10 nM.

### NMZ, but not CMZ, restores LTP in hippocampal slices from APP/PS1 mice

**Fig. 4:**
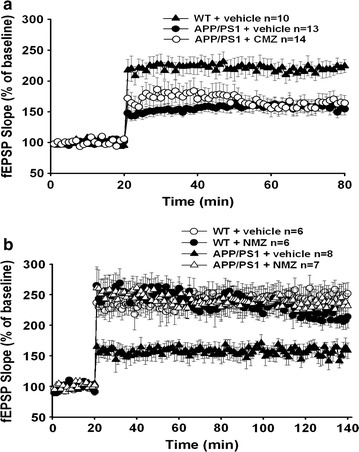
Beneficial effects seen in LTP from NMZ treatment in APP/PS1 mice. **a**, **b** LTP was measured in the CA1 region of hippocampal sections in 4 month old male APP/PS1 mice or littermate controls (n = 5–8) treated with CMZ (**a**) or NMZ (**b**). NMZ showed restoration of LTP in APP/PS1 mice to WT levels, whereas the effects of CMZ were not significant. Statistical significance was analyzed by two-way ANOVA with repeated measures: WT veh (n = 6) vs. WT NMZ (n = 6): F(1,10) = 1.106 p > 0.05 No Sig.; WT veh vs. APP/PS1 veh: F(1,12) = 18.86 p < 0.05 Sig; APP/PS1 veh (n = 8) vs. APP/PS1 NMZ (n = 7): F(1,13) = 17.71 p < 0.05 Sig; WT NMZ vs. APP/PS1 NMZ: F(1,11) = 0.02351 p > 0.05 No Sig

In hippocampal slices from 3-month-old male mice, the effect of NMZ and CMZ on long-term potentiation (LTP) was measured in the CA3-CA1 pathway. After 15 min of baseline collection, drug (100 μM) was added to the bath solution for 5 min prior to induction of LTP using three trains of ten theta bursts, and the resulting fEPSP were recorded in the CA1 area for 120 min. CMZ had no significant effect, whereas NMZ induced a significant increase in fEPSP slope after LTP induction compared to the untreated transgenic, to levels indistinguishable from WT control (Fig. 4). NMZ perfusion of WT hippocampal slices had no significant effect (data not shown). It is important to note that NMZ treatment had no effect on LTP induced in hippocampal slices from WT mice, but restored LTP in slices from APP/PS1 mice. This observation is compatible with the ability of NMZ to activate NO/cGMP/pCREB signaling, when signaling is impaired.

## Discussion

The Amyloid Hypothesis, derived from convergence of genetic, cell biological, and pathological studies [45, 46], states that a primary effect of genetic alterations that cause familial AD is alteration of Aβ production or clearance in a way that promotes its aggregation and accumulation in the brain. However, it remains unclear precisely what role Aβ plays in disease pathogenesis, since Aβ accumulation does not correlate well with the extent of neuronal loss or cognitive dysfunction [47]. Lowering brain Aβ levels has remained the primary target for therapeutic intervention in AD; however, the failure of multiple such therapeutic agents to reach primary endpoints in phase 3 clinical trials [48, 49], emphasizes the urgent need for new approaches. In a substantial population of dementia patients with AD pathology, the correlation of Aβ histopathology with cognitive decline is poor [12, 13], and Aβ-specific therapeutics would be expected to fail in this clinical population. Other aging-associated diseases, such as vascular lesions and diabetes, are also linked to AD pathogenesis [50, 51]. In addition, AD is a multifactorial disease, many contributors to which are common to other dementias and comorbid diseases, for example, glutamate excitotoxicity, oxidative stress, and inflammation [52]. Neuroprotective agents have been targeted at these and other contributors to neuronal loss and we reasoned that one of these, CMZ, would provide a starting point for modest redesign. An anticonvulsant and anxiolytic, CMZ was repurposed in Phase 3 clinical trials as a neuroprotective drug for use in spinal cord injury and ischemic stroke [29, 53, 54], and is prescribed for anxiety and agitation in the elderly [55].

In this context, our redesigned molecule, NMZ, was intended to retain the beneficial therapeutic properties of CMZ, a positive allosteric modulator of GABA_A_ function, which potentiates the function of the inhibitory neurotransmitter GABA in the brain [56, 57] and therefore attenuates the glutamate-induced excitotoxic cascade leading to mitochondrial damage and neuronal loss [26–28]. CMZ potentiates GABA and muscimol agonism at the GABA_A_ receptor without evidence for modulation of levels of GABA itself; opens neuronal Ca-dependent Cl¯ channels enhancing inhibitory neurotransmission; is a direct GABA_A_ receptor agonist at concentrations 100-fold than those required to potentiate GABA function (30 μM); is not a ligand for the GABA_B_ nor benzodiazepine receptors and; potentiates the actions of glycine on inhibitory neurotransmission [38, 39]. NMZ was shown to potentiate the actions of GABA at the α_1_β_2_γ_2_ GABA_A_ receptor without having direct actions on ion current gated by this receptor. Loss and recovery of righting reflex provides a measure of profound, reversible sedation; whereas latency to fall from the accelerating rotarod provides a measure of transient sedation. These tests clearly demonstrated that CMZ and NMZ both cause transient sedation, but that CMZ is significantly more potent. The sedative actions of NMZ further support the conclusion that NMZ retains the activity of CMZ at the GABA_A_ receptor. Sedation itself is not directly relevant to the proposed beneficial actions of NMZ in dementia and was observed at a dose 200-fold higher than that required to counter memory loss.

Selective pharmacological activation of GABA_A_ receptors has been shown to provide neuroprotection against A*β* mediated toxicity [58–60], and the GABA-potentiating [60, 61] and anti-TNF-α [62, 63] properties of CMZ are of clinical utility in AD; nevertheless, we hypothesized that it was essential also to address synaptic dysfunction [64, 65]. Several studies indicate that restoration of synaptic failure can be achieved through CREB activation [66, 67]. The coupling of CREB activation to NO/cGMP signaling in the hippocampus [34, 68, 69], inspired the design of NMZ [70, 71]. We and others have demonstrated that agents activating NO/cGMP/CREB can significantly improve LTP and cognitive function in mice and rats [72, 73], including in studies of scopolamine-induced amnesia [74, 75]. NMZ treatment was able to reverse deficits in working memory in the STPA task induced by blockade of muscarinic and NMDA receptors, in addition to deficits induced by diazepam and inhibition of nitric oxide synthase (NOS). More importantly, by varying the time of drug administration, we demonstrated that NMZ reverses cholinergic deficits administered 90 min after training, functioning via memory consolidation. The cholinergic hypothesis remains relevant to age-related dementia and AD [76, 77].

PK/PD relationships were also obtained for NMZ actions on working memory by varying the time of drug administration. Administration of NMZ i.p. at 1 mg/kg and p.o. at 20 mg/kg/day gave approximate peak and trough brain concentrations of 80 and 0.5 nM, respectively. The loss of procognitive activity corresponded to brain concentrations of NMZ falling below 1 nM during training. Oral delivery of NMZ at 20 mg/kg/day was procognitive and yielded a mean brain concentration of 0.73 nM. The measured brain/plasma ratio approached unity and the observed sedative activity required concentrations of NMZ in brain tissues reaching 10 µM. The high potency of NMZ, in vivo, for cognition enhancement (~1 nM) and the large separation of sedative and procognitive actions (~10^5^-fold) is encouraging for clinical efficacy and safety. In accord with predictions for improvement of the function of CMZ by incorporating NO/cGMP/CREB signaling in NMZ, perfusion of hippocampal slices from transgenic APP/PS1 mouse with NMZ, but not CMZ, rescued LTP in this model of AD.

## Conclusions

In summary, NMZ, an orally active, brain-bioavailable small molecule represents a novel therapeutic approach to dementia and AD. The re-engineering of a clinical neuroprotective agent, CMZ, to address synaptic dysfunction by incorporating the capacity to activate NO/cGMP/CREB signaling was achieved without loss of the GABA_A_ potentiating actions of the parent drug. The increasing recognition that AD and age-related dementia may have underlying mixed pathology, supports a small molecule systems pharmacology approach to neurodegeneration and dementia.

## Methods

### Test compound

The synthesis of NMZ followed standard acidic nitration procedures from 4-methyl-5-(2-hydroxyethyl)-1,3-thiazole, followed by salt formation with HCl and crystallization. Purity as assessed by chromatography and spectroscopy was greater than 98 %. NMZ is a crystalline white solid: mp 107–108 °C (d) [72].

### Animals

All animal care and procedures were conducted with approved institutional animal care protocols and in accordance with the NIH Guide for the Care and use of Laboratory Animals. All animal protocols were approved by the University of Illinois at Chicago Institutional Animal Care and Use Committee and cognate committees at Columbia University. Double transgenic mice (APP/PS1) expressing both the human APP_695_^K670N/M671L^ and PS1^M146L^ mutations were compared with wild type littermates and housed at Columbia University [78]. Male C57BL/6 mice were obtained from Charles River Laboratories (Wilminton, MA, USA) and used for bioavailability studies (3 months of age), and STPA and sedation studies (5–8 months of age), and housed at UIC. Food and water were available ad libitum, except during the vehicle and drug treatment periods using either drinking water or hydrogel. Hydrogel preparation was as previously described [44]. Stability of NMZ in hydrogel or drinking water was assayed, showing no significant reduction of drug concentration. For electrophysiology experiments, *Xenopus laevis* toads, were used as the source of oocytes for engineered expression of α2β2γ2 GABA_A_ receptors, were obtained from Xenopus One (Ann Arbor, MI, USA). All animal maintenance and surgical procedures on *X. laevis* conformed to UIC institutional policies (BRL protocol 13-125) and to the Statement for the Use of Animals in Ophthalmic and Vision Research adopted by the Association for Research in Vision and Ophthalmology.

### Electrophysiology

*GABA*_*A*_*potentiation* Experiments were conducted on *Xenopus laevis* oocytes expressing α_1_β_2_γ_2_ GABA_A_ (rat α_1_, rat β_2_ and human γ_2S_) receptors using methods previously described [31]. Briefly, oocytes were prepared by cRNA injection and studied by two-electrode voltage-clamp recording. Oocytes were superfused with Ringer solution at a rate of ~1 ml min^−1^. Glass micropipettes for oocyte recording were prepared with a resistance of 1–10 MΩ when filled with 3 M KCl. Test solutions were delivered via multiple channels from separate reservoirs by a gravity flow system. Membrane current data were obtained using Clampex 8.2 and analyzed using Clampfit 10.0 (Axon Instruments) and OriginPro7.5 (OriginLab Corporation, Northampton, MA). *LTP* Mice were sacrificed by cervical dislocation followed by decapitation and immediate preparation of 400 μm hippocampal slices. Slices were maintained at the interface between a continuous perfusion of artificial erebrospinal fluid (aCSF) and an atmosphere of 95 % O_2_ and 5 % CO_2_. After at least 1 h of recovery, stimulation electrodes were placed in *stratum radiatum* of field CA1 to activate Schaffer/commissural fibers and glass recording micripipettes were placed in the same layer to record field excitatory postsynaptic potentials (fEPSPs) in the synaptic zone. Stimulus intensity was set to evoke submaximal fEPSP and continuously monitored at 20–60 s intervals for at least 15 min to establish a stable baseline. As described previously [79], LTP was induced by theta-burst stimulation (TBS), consisting of four-pulse bursts of high frequency (100 Hz) stimulation, repeated ten times at 5 Hz. APP/PS1 hippocampal slices were perfused with test compound (100 μM) for 20 min before inducing LTP. The results were expressed as mean ± SEM

### Behavior

*Evaluation of sedation by rotarod* Sedation was measured using the accelerating rotarod test as previously described [31, 80]. Briefly, animals were placed, up to four at a time, on individual rods within a lane. Latency to fall was detected with a 0.1 s temporal resolution by a series of photocells located above the rotating rod in the apparatus. Mice were trained for 3–5 days on the rotarod prior to testing until the mean latency to fall reached 100 ± 10 s. Each training session started with a 30 s trial on a non-rotating rod, followed by 60 s rotating at constant speed (4 rpm). Animals were then subjected to open ended time trials on the accelerating rod (4–40 rpm within 120 s), and the latency to fall off the rotorod was recorded. Each animal was given four trials per day to obtain a baseline level of performance. After drug administration, mean latencies to fall off were recorded at different time points (10, 30, and 60 min post injection). *Loss of righting reflex* In LORR test, after drug administration, animals are placed on their back on a flat tissue paper laid surface maintained at around 30 ℃. The time to loss of the righting reflex is recorded. Loss is considered to have occurred if the animal remains on its back for more than 30 s. Righting reflex is considered to be regained when the mice were able to successfully right themselves with all four paws touching the floor, twice within 20 s [81]. *Step through passive avoidance (STPA)* STPA has been widely used to test long-term working memory and was performed as we have previously described [82–85]. Each animal was given two ip injections: (1) Amnesic agent (scopolamine 1 mg/kg, MK-801 0.1 mg/kg, diazepam 0.5 mg/kg, L-NAME 50 mg/kg) or saline were given 30 min prior to training plus NMZ or saline in the time and delivery method as indicated in Fig. 2a. Briefly, mice were habituated in a light/dark box prior to training in the same box, in which mice placed in the light compartment received an electric shock on entering the dark compartment (0.5 mA, 60 Hz for 2 s). This training was repeated until latency to enter the dark side reached 300 s. At 24 h and/or 48 h post-training, animals were individually placed in the light compartment and the latency to enter the dark compartment was recorded with a 300 s cutoff.

### Brain bioavailability

Three-month-old male C57BL/6 mice were administered NMZ by i.p. injection plus hydrogel (1 mg/kg i.p., 20 mg/kg/day oral) or i.p. injection (50 mg/kg). At the appropriate time point, mice were sacrificed using CO_2_ asphyxiation. Blood was immediately collected from the dorsal aorta in 1 mL K3EDTA tubes (Greiner Vacuette) and kept on ice. After centrifugation at (3200×*g* for 20 min at 4 °C), the plasma supernatant was collected and immediately processed for analyses. Remaining plasma was stored at 80 °C. Following blood collection, each mouse was intracardially perfused with ice-cold PBS buffer (pH 7.4) and was decapitated. Brain was separated by hemisphere and half hemisphere was immediately processed for analyses while the other half was flash frozen with liquid nitrogen to be stored at −80 °C. Briefly, the reconstituted brain and plasma samples were analyzed after addition of the internal standard. Each sample was analyzed in triplicate and each sample set was analyzed with a set of calibration standards. Peak areas for analytes and standards were calculated and the amount of each compound in each sample was determined using the calibration curves (Additional file 1). We have previously measured plasma pharmacokinetics of NMZ in rats, reporting: AUC-determined oral bioavailability of 20.5 and elimination half-lives of 32.6 min (p.o.) and 19.0 min (i.p.) [72].

### Statistics

The data were reported as the mean ± SEM or SD using student’s *t* test and/or one-way ANOVA analysis with Tukey’s multiple comparison test, or ANOVA with repeated measures, by using Graph-Pad Prism version 4.00 for Windows, GraphPad Software.

## Additional file

10.1186/s12868-015-0208-9 Supplementary Pharmacokinetic Experimental Details.

## Abbreviations

cGMP: cyclic guanosine monophosphate
CMZ: chlormethiazole (also clomethiazole)
CREB: cAMP response element-binding protein
fEPSP: field excitatory postsynaptic potential
GABA: gamma-aminobutyric acid
HMZ: 5-(2-hydroxyethyl)-4-methylthiazole
LORR: loss of righting reflex
LTP: long-term potentiation
MCI: mild cognitive impairment
NMDA: *N*-methyl-d-aspartic acid
NMZ: 4-methyl-5-(2-(nitrooxy)ethyl)thiazol-3-ium chloride
NOS: nitric oxide synthase
pCREB: phosphorylated cAMP response element-binding protein
sGC: soluble guanylyl cyclase
STPA: step-through passive avoidance
