# Supplementary material for: Re-engineering a neuroprotective, clinical drug as a procognitive agent with high in vivo potency and with GABAA potentiating activity for use in dementia
Source: BMC Neurosci. 2015 Oct 19;16:67. doi: 10.1186/s12868-015-0208-9 (PMC4612403; doi:10.1186/s12868-015-0208-9)
Supplement: Supplementary file 1 — 10.1186/s12868-015-0208-9 Supplementary Pharmacokinetic Experimental Details. [file 12868_2015_208_MOESM1_ESM.pdf]

# Supplemental Methods

**Test compound extraction:** For the extraction from plasma, 350 µL ice- cold methanol, and the recovery standard (0.50 pmols) were added to the fresh plasma sample (100 µL) immediately after collection. The samples were then vortexed for about 30 sec, centrifuged for 15 min at 14000 rpm at 4°C prior to extracting the supernatant. Another 100 µL of ice-cold methanol was added to the residual fraction, vortexed, centrifuged, and the supernatant was collected and pooled with the previously-collected supernatant fraction.

For the extraction from brain tissues, 650 µL ice-cold methanol and the recovery standard (1.5 pmols) were added to each half-hemisphere brain sample (~160 mg) immediately after collection. The samples were thoroughly homogenized using a hand-held-pestle grinder (Fisher Scientific, Hudson, NH) with a disposable pellet pestle (Kimble Chase, Vineland, NJ). The homogenized samples were centrifuged for 15 min at 14000 rpm at 4 °C prior to extracting the supernatant. Another 350 µL of ice-cold methanol was added to the residual pellet, homogenized, centrifuged, and the supernatant was collected and pooled with the previously collected fraction of supernatant.

The collected supernatant samples from brain tissues and plasma were dried with gentle nitrogen blow-down, reconstituted with methanol (100 µL), and stored at -20 °C until analyses. LCMS grade solvents and low retention Eppendorf tubes (Thermo Scientific, Vernon Hills, IL) were employed for sample preparation.

**LC-MS/MS method development:** The LC–MS/MS analysis was performed using an Agilent 1200 liquid chromatographic system attached to API 3000 triple quadrupole mass spectrometer equipped with an electrospray ionization (ESI) source. Waters XBridge C18 (3.5 µm, 3.00 x 100 mm) column was used to achieve the separation at a constant flow rate of 300 µL/min of mobile phase which is water with 15% methanol (A) and methanol with 20% acetonitrile (B). In the solvent program, B was ramped from 28% to 48% within 5 min, then ramped up to 95% in 7 min and was maintained at the final condition for another 3 min. Multiple reaction monitoring (MRM) channels of 189 $\rightarrow$ 113, 144 $\rightarrow$ 113, 220 $\rightarrow$ 189, 238 $\rightarrow$ 207 were selected to detect NMZ, HMZ , recovery standard, and internal standard, respectively in the positive ESI mode.

Standard curves were prepared for NMZ (2.75-88.0 nM), HMZ (0.75-24.0 nM), and for the recovery standard (0.625-20.0 nM) while using the internal standard. The stock and the working solutions were prepared in MS grade methanol. The reconstituted brain and plasma samples were diluted with methanol and were analyzed after adding the internal standard. Each sample was analyzed in triplicate and each sample set was analyzed with a set of calibration standards. Peak areas for analytes and standards were calculated and the amount of each compound in each sample was determined using the calibration curves.
